# Fatal massive hemolysis caused by immunoglobulin M anti‐c antibody in a patient with newly diagnosed B‐cell acute lymphoblastic leukemia: a case report

**DOI:** 10.1002/ccr3.1534

**Published:** 2018-04-17

**Authors:** Carlos Galvez, Abdulrahman Abutaleb, Wade T. Iams, Paul F. Lindholm, Hau C. Kwaan

**Affiliations:** ^1^ Department of Internal Medicine Northwestern University Feinberg School of Medicine Chicago Illinois; ^2^ Division of Hematology/Oncology Northwestern University Feinberg School of Medicine Chicago Illinois; ^3^ Department of Pathology Northwestern University Feinberg School of Medicine Chicago Illinois

**Keywords:** Anti‐c antibody, hemolytic transfusion reaction, IgM

## Abstract

Delayed hemolytic transfusion reactions (DHTRs) occur secondary to slow, mild IgG‐mediated processes against minor red blood cell antigens. Herein, we report the case of a rapidly fatal alloimmune anti‐c IgM‐mediated hemolysis, a rare, previously undescribed, pathophysiologic scenario. Early recognition of such phenomena can expedite supportive measures and optimize patient outcomes.

## Description of Case/Case Report

The patient is a 71‐year‐old female who presented with generalized weakness, fatigue, night sweats, and a five pound weight loss over 2 weeks. A complete blood count (CBC) on admission showed pancytopenia with a white blood cell count (WBC) of 2.2 K/UL (59.1% lymphocytes, 15.6% monocytes, and 21% neutrophils), hemoglobin (Hgb) 8.7 g/dL, and platelets of 25 K/UL. She had a normal CBC 2 months prior to presentation. A bone marrow biopsy was performed on admission which confirmed the diagnosis of B‐cell acute lymphoblastic leukemia (B‐ALL) with 90% blasts. Within 48 h of presentation, the patient's Hgb decreased, and she received one unit of leukoreduced (LR), irradiated (IRR) packed erythrocytes. Her pancytopenia was managed in standard fashion for anemia associated the hematologic malignancies with red cell replacement. She had no immediate reaction to blood transfusion, which would typically be mediated by hypersensitivity to circulating factors in the transfusate rather than an immune response directed at transfused erythrocytes. Noninfectious transfusion reactions are a set of well‐documented adverse events that can occur during or shortly after infusion of blood products. The most common transfusion reactions are febrile nonhemolytic transfusion reactions (FNHTR), which are also the least serious and can be made less frequent by leukoreducing blood products prior to infusion 1, 2.

**Figure 1:**
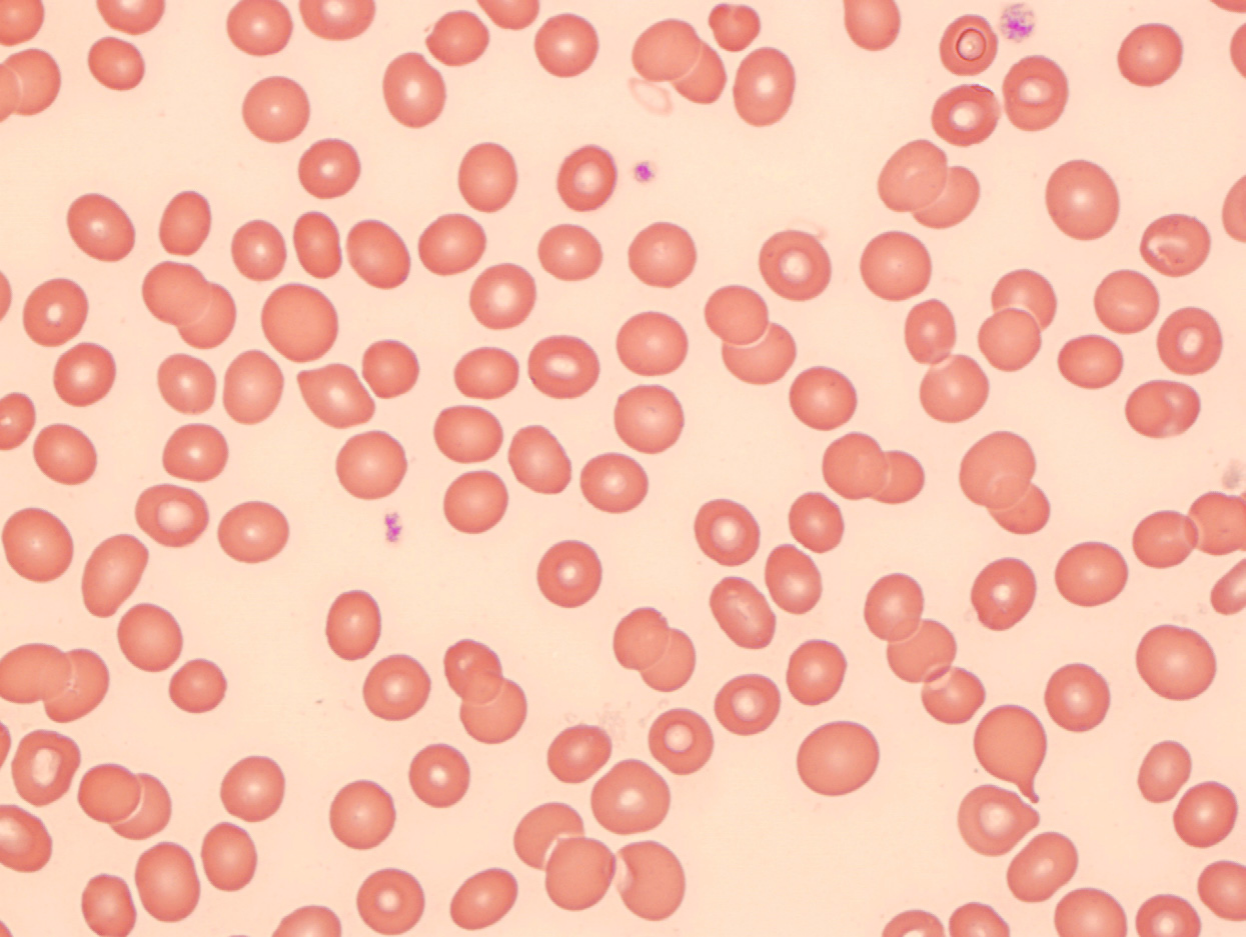
Peripheral blood smear from admission showing a mild hypochromic anemia with no spherocytes.

On the third day of hospitalization, the patient received an additional unit of erythrocytes for a Hgb of 7.2 g/dL. The erythrocytes were cross‐matched, LR, and IRR. At the time of receipt of her second unit of erythrocytes, her LDH had decreased from 869 U/L on admission to 470 U/L, a direct antiglobulin test was negative for IgG antibodies, and there was no evidence of red cells with abnormal morphology on her peripheral smear (Fig. 1).

The patient had ongoing anemia secondary to marrow failure at this time, and as the LDH decrease, a dynamic marker in cases of hemolysis had decreased. Furthermore, there were no antibodies present on the surface of erythrocytes, indicating that any immune response was not detected at this time.

On the fifth day of hospitalization, approximately 48 h after her second unit of erythrocyte transfusion, the patient reported increasing abdominal pain and tachypnea. Her Hgb decreased to 6.1 g/dL, and both her LDH and total bilirubin increased (518–882 U/L and 1.4–2.6 mg/dL, respectively). The patient received one unit of LR, IRR, and c antigen‐negative erythrocytes, but she developed hypotension, tachycardia, worsening tachypnea, and hypoxemia over the next six hours. She was intubated, and an arterial blood gas showed a pH of 6.92, partial pressure of CO_2_ of 19 mmHg, partial pressure of oxygen of 243 mmHg, bicarbonate level of 4 mEq/L, and Hgb of 3.5 g/dL. She became increasingly hypotensive requiring four vasopressor agents for cardiovascular support, and she was treated with stress‐dose glucocorticoids.

**Figure 2:**
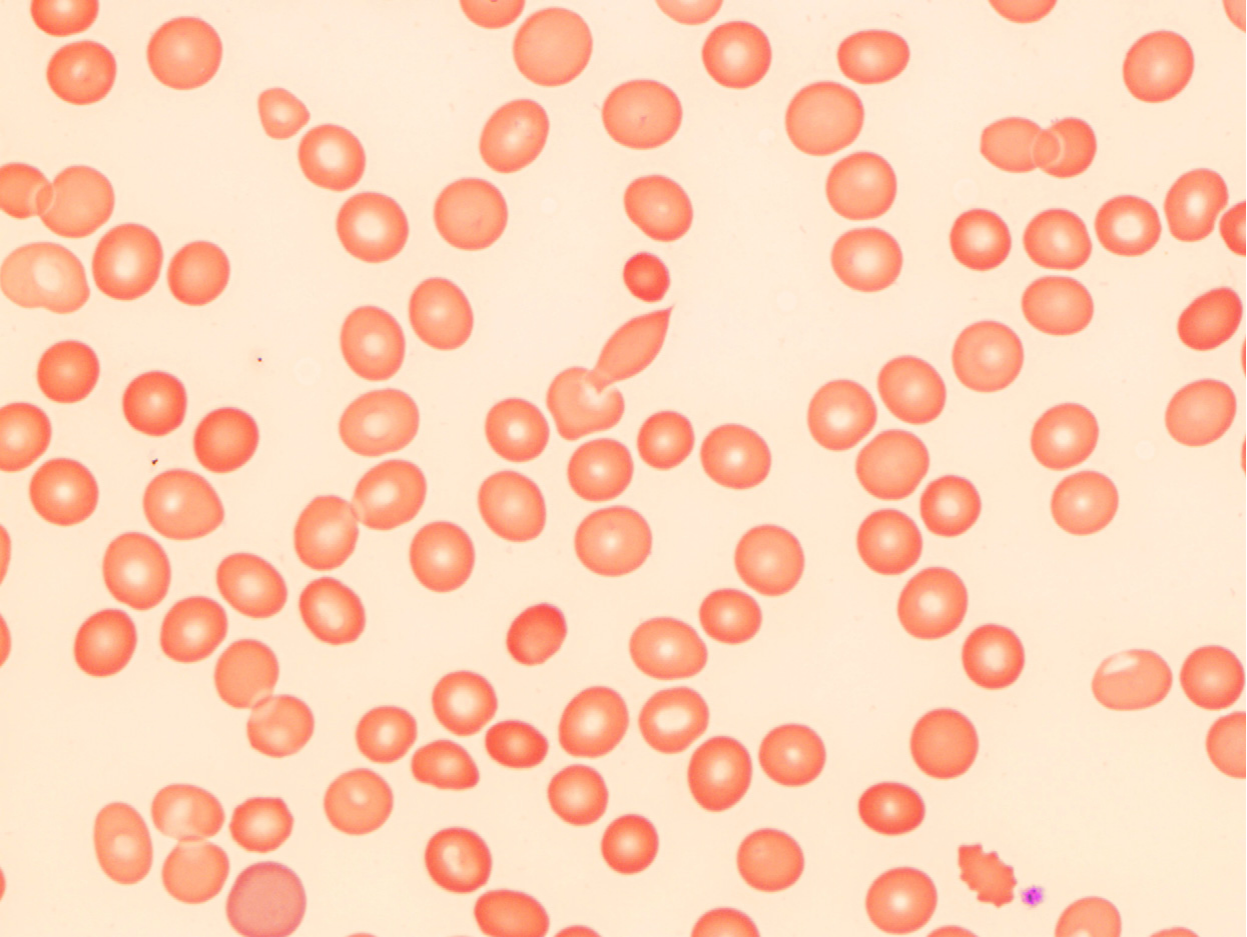
Peripheral blood smear from the day before collapse showing a mild hypochromic anemia with occasional microspherocytes.

The patient exhibited clinical manifestations (tachypnea as compensation for both severe anemia and a severe metabolic acidosis) of a change in her body's response to the marrow failure and acute leukemia. Further evaluation revealed a fall in Hgb 3.5 g/dL (down from 6.6), platelets 13,000/*μ*L (down from 27,000), and fibrinogen 336 mg/dL (down from 615), INR 2.4 (up from 1.4), and aPTT 39.1 sec (up from 30.7). Red cell morphology was significant for marked spherocytosis without mention of schistocytes compared to prior morning's sample with slight spherocytosis and rare schistocytes (Fig. 2). Although disseminated intravascular coagulation (DIC) in the setting of massive hemolysis could not be definitely ruled out, the marked spherocytosis and rare schistocytes were indicative of an immune‐mediated hemolytic process rather than microangiopathic hemolytic anemia (MAHA).

The patient had been started on empiric piperacillin–tazobactam for neutropenic fever on the fourth day of admission but was otherwise not on other medications known to cause significant hemolysis. Penicillins, including piperacillin, are known inducers of hemolytic anemia characterized by IgG‐mediated warm agglutinin disease which improves with prompt discontinuation of penicillin‐containing agents.

Our patient did not have a history of cardiomyopathy nor did she ever endorse localizing infectious symptoms during admission with repeated negative infectious studies including chest X‐ray, urinalysis/urine culture, and three sets of blood cultures. Her catastrophic clinical decompensation was ultimately more consistent with a massive hemolytic event than either one of infectious or cardiogenic etiology. Acute hemolytic transfusion reactions are often severe, and they can be fatal in up to 1 of 100,000 cases 1, 3. In order to avoid hemolytic transfusion reactions, blood products are IgG‐cross‐matched as a standard of care to detect incompatibility due to unexpected alloantibodies 1, 2. However, in delayed hemolytic transfusion reactions (DHTRs), alloantibodies are undetectable until antigen‐positive blood is transfused. Within 5–15 days of blood production infusion, the recipient forms alloantibodies, most commonly against the Rh antigen loci C, D, or E 1, 3. These antibodies subsequently bind to the patient's erythrocytes and future transfused blood products that are alloantigen‐positive. DHTRs are usually IgG‐mediated, mild, and gradual, and the hemolysis occurs extravascularly through splenic sequestration of circulating IgG‐bound erythrocytes. However, if the IgG antibodies bind complement on erythrocytes, severe intravascular hemolysis can occur 1, 4.

In our patient, a second antibody screen for IgG antibodies was performed which was positive for anti‐c antibodies. The direct antiglobulin test was weakly positive for IgG and 1+ for C3 complement, and an IgG red blood cell eluate from the patient reacted with c antigen‐positive blood cells. In addition, the patient's blood reacted to c antigen at 4 degrees Celsius. The reactivity of the anti‐c antibody in the patient's blood was inactivated by dithiothreitol (DTT), indicating that this antibody was of the IgM type.

Understanding potential etiologies of hemolysis, especially in the setting of a possible transfusion reaction, is of critical importance to patient care. FNHTRs mediated by host reactivity to donor plasma components are the most common forms encountered in the clinical setting 1, 2, 3. Hemolysis can be caused by an IgG drug‐induced antibody. The data in our patient, however, indicate that this acute hemolysis was mediated by an IgM antibody to the c antigen, a previously undescribed phenomenon.

The patient was then given two units of c antigen‐negative RBCs that were IgG‐cross‐match‐compatible. She had an appropriate Hgb response from 3.5 to 7.0 g/dL. However, the patient's cardiovascular failure did not reverse, and she developed acute respiratory distress syndrome, disseminated intravascular coagulation, and renal failure requiring dialysis. She died on the sixth day of hospitalization, and no autopsy was performed.

When transfused with c antigen‐negative RBCs that were IgG‐cross‐match‐compatible, the patient exhibited an appropriate hemoglobin response. The novelty of this case is several‐fold, in that for a DHTR, this was extraordinarily severe, rapid, and it hinged on both an unusual antibody type (IgM rather than IgG) to the c antigen 1, 3, 5.

## Discussion

Lymphoproliferative disorders (LPDs) are associated with the development of autoimmune hematologic conditions. Some series have noted that up to 8% of patients with LPDs are diagnosed with an associated autoimmune disorder, and approximately 5% of patients with LPDs develop autoimmune disorders after diagnosis of the LPD^6^. Although the frequency of autoimmune hematologic conditions ranges widely amongst LPDs, the shared pathophysiology is thought to be secondary to systemic immune dysregulation, loss of tolerance to self‐antigens, and production of autoantibodies. As an example, chronic lymphocytic leukemia (CLL) has been associated with autoimmune hemolytic anemia (AIHA) in 11% of patients with advanced stage disease and idiopathic thrombocytopenia (ITP) in 2–3% of patients with early‐stage disease. These autoimmune phenomena in patients with CLL are secondary to the production of IgG (87% of cases) or IgM (13% of cases) autoantibodies 7, 8. Also, cold agglutinin disease (CAD) has been associated with an underlying B‐cell LPD in up to 75% of cases, most commonly via an IgM autoantibody produced in patients with lymphoplasmacytic lymphoma 9.

This is a case of a patient with newly diagnosed B‐cell acute lymphoblastic leukemia (B‐ALL) who had a rapidly fatal IgM‐mediated hemolysis, a result of developing an alloantibody to Rh c red blood cell antigen. The patient's pretransfusion antibody screens were negative, and shortly after the transfusion, the patient's antibody screen became positive for anti‐c. The direct antiglobulin test also became positive for IgG and C3 complement and the RBC eluate contained anti‐c, which had IgM nature, as it reacted at room temperature and was inactivated by DTT. The patient's pretransfusion phenotype later tested negative for c antigen. Taken together, these data indicate that the newly formed anti‐c was an alloantibody with IgM nature. Thorough literature search revealed no analogous cases of a patient with ALL who developed such a reaction.

In our patient, the rapid onset of the reaction led to a fatal outcome. One possibility is that the IgM alloantibody caused erythrocyte agglutination leading to extensive microthrombi formation and the resultant circulatory failure. The phenomenon of erythrocyte agglutination resulting in microthrombi formation is known from the patients with cold agglutinin disease and cryoglobulinemia 10, 11, 12, but it has not been described in the setting of IgM‐mediated hemolysis.

This case provides the first documentation that an IgM‐mediated Rh c antigen‐directed hemolysis can occur. In our case, in the setting of underlying B‐ALL, this reaction was ultimately fatal.

## Conflict of Interest

None declared.

## Authorship

CG, AA, WI, PFL, and HCK: all contributed equally to the writing of this case report.

## References

[1] Dzieczkowski, J. S. , and K. C. Anderson . 2015 Transfusion biology and therapy *in* Kasper D., Fauci A., Hauser S., Longo D., Jameson J. and Loscalzo J., eds. Harrison's principles of internal medicine, 19e. McGraw‐Hill, New York.

[2] King, K. E. , R. S. Shirey , S. K. Thoman , D. Bensen‐Kennedy , W. S. Tanz , and P. M. Ness . 2004 Universal leukoreduction decreases the incidence of febrile nonhemolytic transfusion reactions to RBCs. Transfusion 44:25–29. 14692963 10.1046/j.0041-1132.2004.00609.x

[3] Strobel, E . 2008 Hemolytic transfusion reactions. Transfus. Med. Hemother. 35:346–353. 21512623 10.1159/000154811 PMC3076326

[4] McCullough, J. , M. A. Refaai , and C. S. Cohn . 2015 Blood procurement and red cell transfusion *in* Kaushansky K., Lichtman M. A., Prchal J. T., Levi M. M., Press O. W., Burns L. J. and Caligiuri M., eds. Williams hematology, 9e. McGraw‐Hill, New York.

[5] Molthan, L. , T. J. Matulewicz , B. Bansal‐Carver , and E. J. Benz . 1984 An immediate hemolytic transfusion reaction due to anti‐C and a delayed hemolytic transfusion reaction due to anti‐Ce+e: hemoglobinemia, hemoglobinuria and transient impaired renal function. Vox Sang. 47:348–353. 6438912 10.1111/j.1423-0410.1984.tb04138.x

[6] Diehl, L. F. , and L. H. Ketchum . 1998 Autoimmune disease and chronic lymphocytic leukemia: autoimmune hemolytic anemia, pure red cell aplasia, and autoimmune thrombocytopenia. Semin. Oncol. 25:80–97. 9482530

[7] Dührsen, U. , W. Augener , T. Zwingers , and G. Brittinger . 2008 Spectrum and frequency of autoimmune derangements in lymphoproliferative disorders: analysis of 637 cases and comparison with myeloproliferative diseases*. Br. J. Haematol. 67:235–239. 10.1111/j.1365-2141.1987.tb02333.x 3499930

[8] Mauro, F. R. , R. Foa , D. Giannarelli , S. Coluzzi , F. Mandelli , and G. Girelli . 2000 Autoimmune hemolytic anemia in chronic lymphocytic leukemia: clinical, therapeutic, and prognostic features. Blood 95:2786–2792. 10779422

[9] Berentsen, S. , E. Ulvestad , R. Langholm , K. Beiske , H. Hjorth‐Hansen , W. Ghanima , et al. 2006 Primary chronic cold agglutinin disease: a population based clinical study of 86 patients. Haematologica 91:460–466. 16585012

[10] Kwaan, H. C. 2011 Microvascular thrombosis: a serious and deadly pathologic process in multiple diseases. Semin. Thromb. Hemost. 37:961–978. 22198861 10.1055/s-0031-1297375

[11] Kwaan, H. C. , and J. Wang . 2003 Hyperviscosity in polycythemia vera and other red cell abnormalities. Semin. Thromb. Hemost. 29:451–458. 14631544 10.1055/s-2003-44552

[12] Ruch, J. , B. McMahon , G. Ramsey , and H. C. Kwaan . 2009 Catastrophic multiple organ ischemia due to an anti‐Pr cold agglutinin developing in a patient with mixed cryoglobulinemia after treatment with rituximab. Am. J. Hematol. 84:120–122. 19097173 10.1002/ajh.21330

